# Improvement of Eco-Efficiency in China: A Comparison of Mandatory and Hybrid Environmental Policy Instruments

**DOI:** 10.3390/ijerph15071473

**Published:** 2018-07-12

**Authors:** Zifeng Liang, Manli Zhang, Qingduo Mao, Bingxin Yu, Ben Ma

**Affiliations:** 1 Political Science and Public Administration School, Shandong University, Qingdao 266000, China; 201505031055@mail.sdu.edu.cn (Z.L.); zhangmanli@mail.sdu.edu.cn (M.Z.); mqd0532@mail.sdu.edu.cn (Q.M.); 201500032059@mail.sdu.edu.cn (B.Y.); 2 Center for Crisis Management Research, School of Public Policy & Management, Tsinghua University, Beijing 100084, China

**Keywords:** eco-efficiency, mandatory environmental policy instruments, hybrid environmental policy instruments, voluntary environmental policy instruments

## Abstract

China’s environmental problems have long been criticized. The Communist Party of China (CPC) and the government have increasingly paid attention to developing environmental protection and included the construction of an ecological civilization in the “Five-in-One” development strategy. The improvement of regional eco-efficiency is an important way to realize the coordinated development of the entire society, and environmental policy instruments are a powerful means to enhance regional eco-efficiency. This paper categorizes environmental policy instruments into mandatory, hybrid, and voluntary types. Based on panel data from 31 provinces in China from 2005 to 2015, the paper discusses the impact of environmental policy instruments on regional eco-efficiency and the means of the impact. The research shows that (1) mandatory and hybrid environmental policy instruments play a significant role in promoting regional eco-efficiency, while the role of voluntary instruments is not significant in promoting regional eco-efficiency; (2) hybrid and mandatory environmental policy instruments have negative interactions; and (3) the level of economic development will positively affect the role of hybrid environmental policy instruments in promoting regional eco-efficiency but negatively affect the role of mandatory instruments in promoting regional efficiency.

## 1. Introduction

For the past 40 years of reform and opening up, China’s economy has maintained rapid growth. However, China’s unreasonable economic structure, inefficient use of resources, and severe environmental pollution have become important factors that have constrained the long-term healthy development of China’s economy. In 2017, China’s GDP reached 82.71 trillion yuan, which was an increase of 6.9% over the previous year and accounted for 15% of the global GDP. However, China’s steel consumption accounts for more than 40% of the global GDP; coal and oil consumption account for more than 80% of its total energy consumption [1,2,3], which is 20% of the global consumption of coal and oil [4] and is thus a great waste of resources. China’s environmental problems have also become increasingly prominent. In 2016, the total amount of sewage discharged in China was as high as 71.11 billion tons, and the total amount of sulphur dioxide emissions was as high as 11.5286 million tons. Of the 338 cities in China, only 84 cities (24.9%) had met the environmental air quality standards, and the average number of days in which the amount of pollution exceeded the pollution standard as a proportion of the total was 21.2% [5,6]. Of the 474 cities (districts and counties) in which precipitation monitoring was conducted, the cities experiencing acid rain with an average annual precipitation pH below 5.6 represented 19.8%, and the average acid rain frequency was 12.7% [7]. According to calculations by the World Bank, the Chinese Academy of Social Sciences, and the Ministry of Environmental Protection of the People’s Republic of China, China’s annual economic losses caused by environmental pollution account for approximately 10% of its GDP, with major negative impacts on social and economic development, production and the lives of its people. To transform the current model of economic development, which comes at the expense of the excessive consumption of resources and environmental pollution, and to achieve sustainable development of the economy, China must strengthen its environmental management [8,9]. Environmental governance is a global problem. To better cope with its environmental problems, China is using the international community and agreements to combat environmental pollution with, for instance, the funding that it received via the Kyoto protocol and the Paris agreement, through which China decided to make more serious commitments with regard to global warming. Further, China has put these commitments into practice [10].

To address the harm caused by the excessive consumption of resources and excessive environmental pollution, the CPC and the government have put environmental protection and ecological construction on the agenda. The report of the Eighteenth National Congress of the Communist Party of China explicitly stated that “Ecological civilization construction and construction of ecological civilization are a long-term plan for the well-being of people and the future of the nation. Faced with the severe situation of tight resource constraints, serious environmental pollution, and degraded ecosystems, we must establish the concept of ecological civilization that respects nature, conforms to nature, and protects nature, place ecological civilization construction in a prominent position, and integrate it into economic construction, political construction, cultural construction, social construction in all aspects and throughout the entire process”. The Chinese government has promulgated a series of laws, including the Law of the People’s Republic of China on the Prevention and Control of Environmental Pollution by Solid Waste (2004), Law of the People’s Republic of China on Conserving Energy (2007 Revision), Water Pollution Prevention and Control Law of the People’s Republic of China (2008), and Atmospheric Pollution Prevention and Control Law of the People’s Republic of China (2015 Revision), to protect the environment. Since 2008, the Ministry of Environmental Protection of the People’s Republic of China has formulated many pollutant discharge standards for industries, including the lead and zinc industry (GB25467), the water discharge associated with the steel industry (GB13456), and the petroleum refining industry (GB31570). These new laws and regulations have forced Chinese enterprises and local governments to treat environmental pollution seriously, and China’s environmental governance is gradually changing. In addition, China has used multiple environmental policy instruments, including the “three-simultaneity” system based on administrative coercive measures, a market-based “discharge charges” system, and voluntary public environmental protection participation to improve eco-efficiency. Specifically, the “three-simultaneity” system refers to the fact that the facilities for preventing and controlling pollution in construction projects must be designed, constructed and put into operation in addition to the main projects. The “discharge charges” system refers to the system that charges polluters who exceed discharge standards according to the relevant regulations. Further, voluntary public environmental protection participation refers to citizens participating in environmental volunteering and promoting environmental awareness to protect the environment. These environmental policy instruments are powerful tools for tackling environmental problems.

Because of the regional nature of environmental problems, local governments recognize and pay attention to environmental problems in different ways such that environmental protection departments in various regions make different choices and implement environmental policies differently. In 2017, the Ministry of Environmental Protection of China conducted a large-scale supervision of environmental protection. In response to the supervision, mining in many quarries was stopped in Shandong, Hebei, Shanxi, and other provinces. At the beginning of 2018, there were fewer haze days in certain provinces than there had been in previous years. Beijing, Shanghai, Guangdong and other regions have taken the lead in applying a pollutant discharge fee to VOCs (volatile organic compounds), which has played an important role in improving regional environmental performance. Guangxi, Yunnan, Zhejiang, and other provinces have established environmental education centres to enhance the public awareness of environmental protection and improve regional environmental performance. Diversified environmental policy instruments provide a reference for solving the complex environmental problems in China. However, it should be noted that there have been profound differences between the central and local governments in terms of legislation and policy implementation. It is difficult for the macro-policies formulated by the central government to fit the actual local situation, and local governments have huge discretionary power, which leads to inadequate enforcement and poor implementation of government. These problems have affected the environmental governance in China. In addition, the lag and lack of environmental legislation in China has become an important issue in the field of environmental governance. The law prescribes that the maximum amount of fines shall not exceed 30% of the losses caused by pollution accidents. Because of the lack of effective punishment for continuous environmental violations of the law such as continuous excessive drainage, the problems of excessive emission and the low cost of lawbreaking have been difficult to solve. Further, China’s legislation on wetland protection has been in a state of deficiency. These issues have created a stubborn problem for environmental governance in China.

However, how can regional eco-efficiency be measured to fully reflect the regional environmental conditions? Can different environmental policy instruments effectively improve regional eco-efficiency? What are the differences in the effects of different environmental policy instruments? Some scholars have conducted in-depth research on the above issues; however, their studies have mainly focused on how to evaluate regional eco-efficiency and environmental sustainability [11,12,13,14] and on the impact of environmental policy instruments on the industrial “three wastes” [15,16], thus, the means for the impact of China’s environmental policy instruments remains to be further studied.

The main contributions of this article are as follows. First, the eco-efficiency of Chinese provinces is evaluated using the entropy weight method. With Howlett and Ramesh’s classification of policy instruments, environmental policy instruments are categorized into mandatory, hybrid, and voluntary types; then, the impact of the three types of instruments on the eco-efficiency of Chinese provinces is assessed. Second, different instruments focus on different targets. The study found that enterprises, as the main targets for intervention by mandatory and hybrid environmental policy instruments, affect the intervention strategies. According to the economic rationale, enterprises prefer a low-cost strategy; therefore, the resulting overlap of mandatory and hybrid environmental policy instruments has negative interaction effects on the effectiveness of the policy. Third, environmental problems differ across regions; thus, environmental governance must be adapted to local conditions. The introduction of the level of economic development as a moderating variable demonstrates how the mandatory and hybrid environmental policy instruments affect eco-efficiency under different levels of economic development. These research conclusions contribute to the scientific understanding of the effectiveness of environmental policy instruments and provide a theoretical basis and reference for local governments in China to select and optimize policy instruments based on local conditions, improve regional environmental quality, and promote the sustainable and healthy development of the economy.

This paper is organized as follows. Section 2 reviews the literature. Section 3 establishes the research hypotheses and introduces the methodology. Section 4 introduces the data and method. Section 5 presents the results. Section 6 presents the discussion. Section 7 presents the research conclusions.

## 2. Literature Review

### 2.1. Eco-Efficiency

The concept of eco-efficiency was first proposed by Swiss industrialist Stephan Schmidheiny [17] and it was later promoted by the World Business Council (WBCSD). Eco-efficiency refers to the ability of firms, industries, regions, or economies to produce more goods and services with fewer impacts on the environment and less consumption of natural resources, thus bringing together economic and ecological issues [18]. Generally, economic development and environmental protection are, to a certain degree, in opposition. Economic development requires resources from the environment and discharges pollutants into the environment. Especially in industrial societies, economic development is often accompanied by environmental pollution. Some developing countries with low productivity even introduce high-polluting and high energy-consuming enterprises from developed countries to develop their economy at the expense of the environment. In a report by Medocs et al. [19] at the Club of Rome in 1972, resources, environment, and economic development were observed to be mutually restrictive. Therefore, the balance between economic development and regional environmental quality has become an important measurement of the quality of governance. However, the question of how to scientifically and effectively measure eco-efficiency has become a problem for enterprises and governments [20].

**Figure 1:**
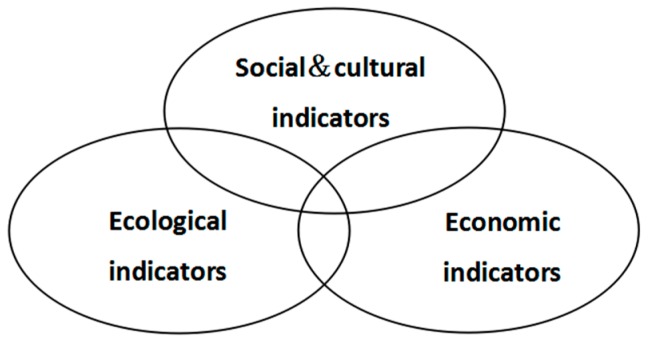
Melanen’s Eco-efficiency Framework.

In 1999, ISO/TC207/SC4 (Technical Committee on Environmental Performance Evaluation and Environmental Management Standardization, Subcommittee of the International Organization for Standardization) formally promulgated the international standard ISO 14031:1999 “Guidelines for Environmental Management—Environmental Performance Evaluation” and the technical report ISO/TR 14032:1999 “Environmental Management Examples of Environmental Performance Evaluations” as a basic framework for measuring eco-efficiency. Currently, there is no consensus on the research regarding methods for measuring eco-efficiency. Researchers differ in their selection of indexes and the determination of index weights. Early researchers in this field include Helge Brattebø of the Norwegian University of Science and Technology and Lv et al. of the Chinese Academy of Sciences. The main contribution of Brattebø [21] was the introduction of social factors into the eco-efficiency measurement system. Brattebø [21] believes that increased attention to social factors can fully and reasonably measure the sustainable development of the society, economy, and environment. Melanen [22] put forward a similar viewpoint; however, Lv et al. [23] believe that compared with economic and environmental indexes, the measurement of a social development index is more subjective, and the concept of eco-efficiency should be based on the analysis of economic and environmental indexes and refer to social development indexes only when necessary. Overall, the research at this stage is limited to the construction of an eco-efficiency framework as shown in Figure 1 and it neither draws a unified conclusion nor involves specific indexes.

To better measure eco-efficiency, the academic community has begun to examine the specific indexes for eco-efficiency and consider different measurement methods. The methods of average weighting, entropy weight, and fuzzy mathematics have been used to measure eco-efficiency. Although scholars choose different indexes and different methods for determining the index weights, the research concept of digitally expressing eco-efficiency lays the foundation for follow-up research.

The current research on eco-efficiency mainly focuses on how to improve a company’s eco-efficiency in consideration of its internal production processes, operational processes, and external regulations and examines the impacts of industrial structure, science and technology investment, urbanization, and foreign direct investment on regional eco-efficiency [24,25]. However, there are few studies on the relationship between environmental policy instruments and eco-efficiency; thus, further study is needed. This paper makes up for this deficiency.

### 2.2. Environmental Policy Instruments

The study of policy instruments has grown with the increasing diversification and complexity of public management entities, networks, and processes of public policy implementation. Policy instruments have been studied throughout the public management system since the reform and the new public management movement.

Policy instrument applications in political science were first proposed by Dahl and Lindblom [26] in “On the Politics adopted by the Modern State-Economic Technology” in the mid-1950s. Due to the powerful explanatory power and application of policy instruments, the theory of policy instruments has been widely applied for environmental protection, waste disposal [27], and land resource management [28]. As environmental governance is valued by governments globally, the research on environmental policy instruments has become more important.

Currently, most research on environmental policy instruments can be placed in the following three research streams. The first is the study of the types of environmental policy instruments. For example, Kemp [29] categorizes environmental policy instruments into command, market, and communication types. Hamilton [30] categorizes them into market-utilized, market-established, environmental regulation, and public mobilization types. Iraldo [31] found that some scholars’ classification methods could be considered one of three types: direct regulation, economic instruments, and “soft” methods. In addition, Howlett and Ramesh [32] likewise observed a trichotomy, categorizing policy instruments by the level of government involvement into the mandatory, hybrid, and voluntary policy instruments. As Howlett and Ramesh present a clear basis and content for their classification of policy instruments, their classification is widely accepted by the academic community. This paper also draws on their classification and divides environmental policy instruments into voluntary, hybrid, and mandatory types.

The second research stream studies the effects of environmental policy instruments on eco-efficiency. For example, Montgomery [33] proved that a tradable emission permit system is the most effective policy tool for optimizing environmental quality. Magat and Viscus [34] demonstrated by applying the least-squares method that environmental regulation can reduce a company’s emissions by 20%. Then, Laplante and Rilstone [35] verified these results via application of the method to a paper mill in Quebec, Canada. Stavins [36] believes that different environmental policy instruments can address different environmental issues. Arimura et al. [37] found that voluntary environmental policy instruments play an important role in reducing pollutant emissions. Zeng et al. [38] proved that mandatory and directly regulated environmental policy instruments have an inhibitory effect on industrial solid waste pollution. These studies mostly explore the impact of one or more specific environmental policy tools on the environment or a particular pollutant; the relationship between environmental policy instruments and eco-efficiency must be further explored.

The third stream of studies considers how environmental policy instruments affect eco-efficiency. The research in this area mainly focuses on two dimensions. One dimension considers the interrelationships among environmental policy instruments and how to match instruments to maximize the effect on eco-efficiency. The other dimension explores the impact of variables other than environmental policy instruments on the effectiveness of the instruments. As early as the 1980s, Doern et al. [32] found that substitution can occur among policy instruments. Xu et al. [39] stressed the importance of the combination of policy instruments. Demirel et al. [33] noted an important link between environmental regulation and self-regulation. Japanese scholar Toshi Arimura [30] used data from Japan to find that voluntary and mandatory policy instruments have a positive interaction. However, there is relatively little research on the relationship between mandatory and hybrid policy instruments. Charles Wolf [34] observed that government intervention in the market may lead to new inefficiencies. This theory is also applicable to the field of environmental management.

In addition, the effect of environmental policy instruments is not uniform across countries and contexts. Norberg-Bohm [35] incorporated corporate innovation into the analytical framework of environmental policy instruments. Zhang et al. [40] believe that environmental information disclosure will affect China’s environmental governance. Peng [36] investigated the effectiveness of environmental regulations. Ren [37] explored the effects of different environmental policy instruments in the eastern, central, and western parts of China. In fact, he introduced the degree of economic development into the analytical framework of environmental policy instruments. Ren [37] found that in the eastern part of China, market-based and voluntary environmental supervision has a significant impact on eco-efficiency and that mandatory environmental supervision is more efficient in the central and western regions. However, Ren’s [37] study does not shed light on how economic development has an impact on environmental policy instruments. Therefore, the subject must be examined in greater depth.

## 3. Hypotheses and Methodology

### 3.1. Hypotheses

Based on the above literature review, this paper proposes the following research hypotheses.

Previous research shows that the three types of environmental policy instruments have different objects of intervention. Mandatory environmental policy instruments mainly aim at enterprises, while hybrid instruments target the public. It is difficult to quantify the effect of the intervention on the public; thus, this paper mainly discusses mandatory and hybrid environmental policy instruments as well as their relationship and roles in promoting eco-efficiency. Based on the above literature review, this paper proposes H1.

**Hypothesis 1** **(H1).** *Mandatory, hybrid, and voluntary environmental policy instruments have a significant role in promoting regional eco-efficiency*.

In the practice of environmental regulation, either the mandatory or hybrid type of environmental policy instrument is generally selected as the main instrument and the other acts as a secondary instrument. If these two instruments are strengthened, the rational enterprise will choose the environmental policy instrument with a lower standard, and the roles of the two instruments will overlap, with a negative interaction between them. Based on this phenomenon, this paper proposes H2.

**Hypothesis 2** **(H2).** *Hybrid and mandatory environmental policy instruments have a negative interaction*.

The effect of environmental policy instruments is influenced not only by the policy combination but also by external factors and regional characteristics. For example, the impact of economic development on environmental policy tools is also reflected in reality. In economically developed regions, such as Shanghai, Guangdong, Jiangsu, and Zhejiang, sewage fee collection institutions have achieved remarkable improvements in eco-efficiency. However, mandatory environmental policy instruments often have little effect on economically developed regions [37]. The industrial structure in economically developing regions is dominated by traditional industries, and mandatory environmental policy instruments tend to have an immediate effect, while elastic hybrid policy instruments are difficult to put into practice and thus have little effect. Based on the above literature review, this paper proposes H3.

**Hypothesis 3** **(H3).** *The degree of economic development will positively affect hybrid environmental policy instruments for the promotion of regional eco-efficiency; however, it will negatively affect the role of mandatory environmental policy instruments in promoting regional eco-efficiency*.

### 3.2. Methodology

We used an economic method to verify the above hypotheses. We adopted the quantitative analysis method and established a panel regression model. We utilized eco-efficiency as the dependent variable and three environmental policy instruments as independent variables. Furthermore, we introduced the regional economic development level as the moderator variable. We used statistical methods to verify the above three hypotheses using Chinese provincial panel data. First, we use the spatial measurement method to conduct a national spatial statistical description of eco-efficiency and generally describe the average situation of eco-efficiency among provinces in 10 years. Second, we identify comprehensive dynamic statistics on changes in eco-efficiency in each province to describe the dynamic evolution of government performance in the past 10 years. Finally, we use the panel data regression method to analyse the cause and effect of eco-efficiency. Among them, the last is a statistical method to verify the above hypotheses directly, and the former two methods supply an overall understanding of eco-efficiency to provide important dependent variable regression analysis data and data characteristics as a whole. In other words, the first two steps are the basis of the last step, although there is no direct verification of the hypotheses. In addition, we developed a unique eco-efficiency evaluation system, which breaks new ground in the research methodology.

## 4. Data and Method

### 4.1. Data Sources

To ensure the accuracy and validity of the data, all the data in this paper (excluding data for Hong Kong, Macao, and Taiwan) come from the corresponding year’s ‘China Statistical Yearbook’, ‘China Financial Statistics Yearbook’, ‘China Environmental Yearbook’, ‘China Environmental Statistical Yearbook’, and ‘China Science and Technology Statistical Yearbook’. All the variables are continuous variables.

Since the implementation of policies generally involves a certain time lag, endogenous problems may arise if there is a correlation and regression analysis of the policy instruments and environmental quality in the same period. Therefore, this paper will address environmental policy instruments as a lag.

### 4.2. Variables

#### 4.2.1. Eco-Efficiency

Given the availability and usability of data, this paper uses the methods of Dong et al. and others to establish an index system that includes eight categories of 17 specific indexes, including three levels of ‘environmental quality, ecological protection, and environmental governance’. These indicators are largely consistent with the results of the previous studies [41,42] and can therefore effectively assess eco-efficiency levels.

Among these indicators, the data of the annual average concentration of sulphur dioxide and the annual average concentration of nitrogen dioxide come from the ‘China Statistical Yearbook’. The data of the annual average concentration of fine particulates (PM10), days when air quality is at or higher than grade two, chemical oxygen demand (COD), rate of industrial waste gas treatment facilities, general industrial solid waste utilization rate, and rate of industrial wastewater treatment facilities come from the ‘China Environmental Statistics Yearbook’. The remaining indexes are drawn from the ‘China Environment Yearbook’.

Index weight assignment is the key to the eco-efficiency evaluation process. The commonly used methods include subjective and objective weighting methods. The subjective weighting method mainly follows the analytical hierarchy process. The objective weighting method includes principal component analysis [43], average weighting, the weighted correlation coefficient method [44], slacks-based measure models [45], super-efficiency DEA models [46,47], and the fuzzy DEMATEL method [48]. However, the subjective weighting method is often subject to the researcher’s personal preference and knowledge level and it cannot guarantee the scientific rigour of the evaluation. The objective weighting method often misses important information because of its simple calculations, and it cannot easily reflect the true level of regional eco-efficiency. Therefore, there is a need to explore new methods for the scientific and effective measurement of the eco-efficiency of provincial administrative units in China.

The concept of entropy was first proposed by Rudolf Clausius to indicate the degree of chaos in the microscopic thermal movement of matter, and the concept can also be used to measure the amount of effective information contained in data and to determine relevant weights. Since the entropy weight method has a strong scientific basis and applicability, it is widely used in assessment in many fields, such as urbanization quality [49], water quality [50], PM2.5 concentration [51], urban sustainable development [52,53,54], the sustainable development potential of an ecosystem [55], soil erosion [56], and the sustainable development capacity of hydropower [57]. The application of the entropy weight method to measure eco-efficiency can overcome the deficiency of existing studies and more scientifically and effectively assess eco-efficiency at a provincial scale. Therefore, this paper uses the entropy weight method to measure the eco-efficiency of provincial administrative units in China [58].

To comprehensively and scientifically measure the eco-efficiency of Chinese provinces, this paper measures eco-efficiency from three dimensions: environmental quality, ecological protection, and environmental governance. Environmental quality includes the discharge of air, water, and solid wastes. Ecological protection includes biodiversity and the town greening condition. Environmental governance includes the conditions of pollution control and environmental regulation. In terms of the index direction, due to the low levels of concentration of sulphur dioxide, carbon dioxide, PM10, and the low level of the emissions intensity of COD, industrial wastewater and industrial solid waste show better eco-efficiency; therefore, the index direction of these indicators is negative. The high levels of forest cover rate, wetlands cover rate, and comprehensive utilization rate of industrial solid waste show better eco-efficiency; therefore, the index direction of these indicators is positive.

The calculation method of eco-efficiency is as follows:

(1) Standardization of all indexes

Higher positive index values are correlated with more desirable values, and lower negative index values are correlated with less desirable values. Therefore, different algorithms are needed to standardize the data for the high- and low-value indexes. The results of the division of positive and negative indexes are shown in Table 1. The specific methods for the standardization of indexes are as follows:

For the indexes where higher values are more desirable, $X_{ij}^{\prime }=\frac{X_{ij}-\min(X_{1j},X_{2j},\cdots ,X_{nj})}{\max(X_{1j},X_{2j},\cdots ,X_{nj})-\min(X_{1j},X_{2j},\cdots ,X_{nj})}$, i=1,2,⋯,n; j=1,2,⋯,m.

For the indexes where lower values are more desirable, $X_{ij}^{\prime }=\frac{\max(X_{1j},X_{2j},\cdots ,X_{nj})-X_{ij}}{\max(X_{1j},X_{2j},\cdots ,X_{nj})-\min(X_{1j},X_{2j},\cdots ,X_{nj})},$ i=1,2,⋯,n; j=1,2,⋯,m.

(2) Calculate the proportion of programme *i* under index *j* to the index

$$P_{ij}=\frac{X_{ij}}{\sum_{i=1}^{n}X_{ij}}\;(j=1,2,\cdots ,m)$$

(3) Calculate the entropy of index *j*

$e_{j}=-k*\sum_{i=1}^{n}P_{ij}\log(P_{ij})$, wherein *k* > 0 and ln is a natural logarithm, *e_j_* ≥ 0. The constant *k* in the formula is related to the number of samples *m*. Generally, set k=1lnm, 0 ≤ e ≤ 1.

(4) Calculate the differential coefficient of index *j*.

For index *j*, greater differences in the index value *X_ij_* are correlated with greater effects on programme evaluation and smaller entropy values.

*g_j_* = 1 − *e_j_*; larger *g_j_* values are correlated with a more important index.

(5) Calculate the weights

$$W_{j}=\frac{g_{j}}{\sum_{j=1}^{m}g_{j}},\;j=1,2,\cdots ,m$$

(6) Calculate the overall score of each programme

$$S_{i}=\sum_{j=1}^{m}W_{j}*P_{ij}\;(i=1,2,\cdots ,n)$$

The weight of each index calculated by the entropy weight method is as follows.

#### 4.2.2. Environmental Policy Instruments

The academic community has not formed a unified standard for the selection of proxy variables for environmental policy instruments. This paper summarizes the proxy variables of environmental policy instruments in Table 2 [58,59,60].

Considering the representativeness of the indexes and the availability of data, this paper has borrowed from the practices of Yu et al. [61]. In this paper, the annual ‘three-simultaneity’ environmental protection investment of various provinces in China represents a measurement index for mandatory environmental policy instruments, the annual sewage charges of various provinces represent a measurement index for hybrid instruments, and the annual number of education activities on social environmental awareness in various provinces represents a measurement index for voluntary instruments.

The mandatory instruments represent the type of environmental policy instrument that is most often used by the government. The tools mainly include direct intervention in the enterprise’s pollution discharge behaviour or the adoption of a ‘shut down, close, transformation’ approach to address environmental pollution issues. A ‘three-simultaneity’ institution is an institution for environmental regulation that originated in China. Article 26 of the Environmental Protection Law of the People’s Republic of China stipulates the following: ‘the facilities for the prevention and control of pollution in construction projects must be designed, constructed and put into operation at the same time as the main project. After the environmental protection department that originally approved the environmental impact report has passed the inspection and acceptance of the pollution prevention and control facilities, the construction project can be put into production or use’. This notion is in line with the characteristics of mandatory environmental policy instruments.

The hybrid instrument is an environmental policy tool with a moderate degree of government involvement that mainly relies on market measures for managing environmental pollution. As early as the last century, British economist Pigou proposed the adoption of a taxation method to overcome the negative externalities caused by corporate sewage discharge. According to the criteria, China’s institution for sewage charges collection meets the characteristics of a hybrid environmental policy instrument.

The voluntary instruments represent the type that requires the lowest level of government involvement. With citizens’ increasing awareness of the environment and rights, voluntary environmental policy instruments play an increasingly important role. Public participation in environmental education can improve the professional knowledge of environmental protection. As a result, the public may pressure companies and local governments to take measures to improve environmental conditions [62]. Therefore, environmental education has the characteristics of a voluntary environmental policy instrument. In addition, green nudges is also a type of voluntary environmental policy instrument. The aim of green nudges is using several behavioural biases to make the behaviour of citizens more environmentally friendly, which is beneficial for improving eco-efficiency [63,64]. Taking the field of energy consumption as an example, Katharina Momsen and Thomas Stoerk [65] found that green nudge increases the percentage of individuals using renewable energy to 44.6% and thus affecting the environment. The formula for the use intensity of environmental policy instruments is shown in Table 3.

#### 4.2.3. Control Variables

The other factors that may affect the local eco-efficiency, including local GDP, industrialization (ind), population (pop), and technology innovation investment intensity (R&D), are included in a regression model as control variables. Of these, the degree of industrialization and the investment intensity of technological innovation are shown in Table 3.

#### 4.2.4. Moderator Variable

The degree of economic development is measured by the per capita disposable income (PCDI).

### 4.3. Calculation Method

The calculation method of all variables is shown in Table 3.

### 4.4. Descriptive Statistics

The descriptive statistics of all the variables involved in this paper are shown in Table 4.

## 5. Results

### 5.1. Overview of Provincial Regional Eco-Efficiency in China

#### 5.1.1. Mean Scale Results of Eco-Efficiency for the Provincial Regions in China

**Figure 2:**
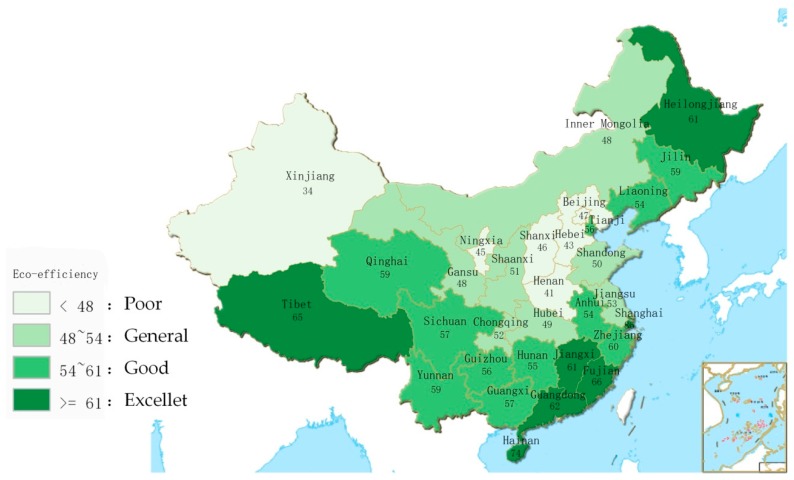
The mean scale results of eco-efficiency for the provincial regions in China.

To visually represent the overall eco-efficiency of the provinces in China over the decade, this paper presents Figure 2, adopting the method of equal number segmentation to classify eco-efficiency into four levels of excellent (≥61), good (54–61), general (48–54), and poor (<48). The excellent level indicates that the environment in the area is unexceptionable. The good level indicates that it still needs improvement in environmental governance. The general level indicates that many problems need to be improved with regard to environmental governance. The poor level indicates that the environmental governance in the area must be improved. As shown in the figure, the provinces with excellent eco-efficiency are mainly concentrated on the southeast coast. In addition, they include Heilongjiang Province and Tibet, which are less industrialized and therefore less polluting. The provinces with good eco-efficiency are Yunan, Guizhou, Sichuan, Jilin, Liaoning, Anhui, and Zhejiang. The provinces with general eco-efficiency include Hubei, Chongqing, Gansu, and Inner Mongolia. The provinces with poor eco-efficiency are mainly concentrated in North China and include Xinjiang, which has a dry climate and relatively low coverage of green land, and Ningxia. Overall, there is a tendency for the eco-efficiency of the coastal areas to be higher than that of the inland areas, and the overall eco-efficiency of the eastern areas is superior to that of the western areas. This finding may be due to industrial optimization, the elimination of outdated production capacity, and the tertiary industry being the mainstay of the eastern region. The industrial transfer in the western region has attracted high-polluting and high energy-consuming enterprises that have been phased out in some eastern regions, yielding lower eco-efficiency in the western region than in the east. In addition, the eastern coastal areas are humid and vegetation rich, and the pollutants thus spread easily. With the developed economy and environmental investment, eco-efficiency in this area is often excellent or good. The central provinces have a dry climate, sparse vegetation, lagging economic development, and less investment in environmental governance; thus, the eco-efficiency in this area is often general or poor.

#### 5.1.2. Radar Chart of Environmental Eco-Efficiency in China

**Figure 3:**
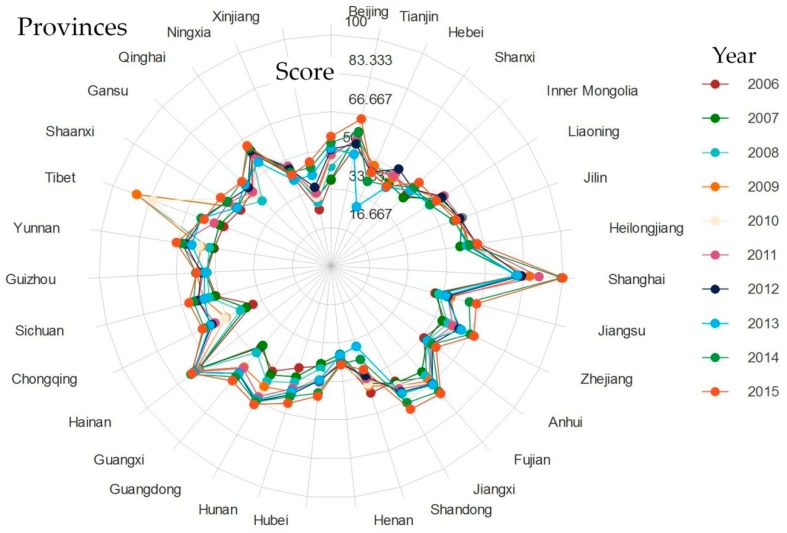
The radar chart of environmental eco-efficiency in the Chinese provinces from 2006 to 2015.

To visually represent the changes in the eco-efficiency of the provinces in China over the decade, this paper presents Figure 3. The figure shows that the eco-efficiency of Shanghai is significantly higher than that of other provinces, and the high level of eco-efficiency in Shanghai is closely related to the large area of wetland and the intensity of investment in environmental governance. The high level of eco-efficiency in Hainan, Fujian is closely related to a high forest cover rate and a high proportion of nature reserves.

The eco-efficiency of Henan remains at a relatively low level over the 10-year period. Although the level of eco-efficiency in Xinjiang remains low, it steadily increases over the period. However, the eco-efficiencies in Shanxi, Inner Mongolia, and the three northeast provinces remains at a moderate level, with relatively small changes. The eco-efficiencies of Hunan, Hubei, and Guangdong also begin at a medium level but undergo major changes, showing a growth trend. The overall change in environmental performance in the remaining the provinces is small. On the whole, since China’s environmental protection legislation has gradually improved, the implementation of environmental protection policies has strengthened, and the Chinese public’s awareness of environmental protection has also grown; however, the eco-efficiency of various provinces in China could still improve.

### 5.2. Panel Regression Model

The contribution of this paper with regard to the conceptual model is to promote research on the relationship between environmental policy instruments and eco-efficiency in the context of Chinese practice. By introducing the moderator variable ‘degree of economic development’, this paper proves that different types of environmental policy instruments have very different levels of influence on the eco-efficiencies in different economic development areas. This provides a useful complement to the environmental policy instruments research. The specific panel regression model is shown in Table 5.

## 6. Discussion

In the process of studying China’s eco-efficiency and environmental policy instruments, the 31 provincial-level administrative units in the country are the research subjects. The 31 provincial administrative units are assumed to represent almost the entire population. At the same time, within the samples, the unobserved traits, such as the qualities of the human capital, resource endowments, and environmental endowments, are assumed to be fixed; thus it is appropriate to use a fixed effects model. However, to ensure statistical rigour, the Hausman test was performed on all the regression models in this paper. If the Hausman test has a *p*-value of less than 0.1, then H0, which states that ‘random effects, i.e., individual effects and interpretations, exist’ is rejected. The fixed effects model is therefore more appropriate, whereas if the *p*-value is greater than 0.1, the random effects model is considered to be more appropriate. The Hausmann test applied to the eight regression models in this paper yields *p*-values less than 0.05; thus, choosing a fixed effects model is appropriate.

Models 1 and 2 show that mandatory and hybrid environmental policy instruments play a significant role in promoting regional eco-efficiency. Model 3 shows that voluntary instruments have a catalytic effect on regional eco-efficiency; however, the effect is not significant. Thus, H1 is partly proved. This result confirms the findings of previous research [34,35,66]. However, the conclusions are not consistent with those of Arimura et al. [38]. This result may be because although China has a socialist market economy, the market economy has emerged from a planned economy, and the traditional intervention of administrative directives in all corners of the society continues to this day. The pattern of strong state-weak societies has caused administrative power to expand, and this tradition has also extended to the field of environmental governance. Therefore, the socially led voluntary environmental policy instruments fail to achieve their desired effects. As a representative of China’s hybrid environmental policy instruments, the sewage charge collection system also relies on administrative power for implementation. Although the degree of involvement of the national government is lower in hybrid environmental policy instruments than in mandatory instruments, hybrid instruments still require ‘administrative intervention’. Therefore, the empirical study shows that only mandatory and hybrid environmental policy instruments have a significant role in promoting regional eco-efficiency, and voluntary instruments have no significant role. However, it should be noted that both the environmental education and ENGO play more important roles in environmental governance in both China and other countries. China’s NGOs actively participate in international forums, such as under the UNFCCC or other initiatives, and a more frequent collaboration and coordination between the State and NGOs has begun in China. Since 2008, the China Institute of Public and Environmental Affairs and the America Natural Resources Defense Council have developed and published a ‘pollution information transparency index’ in 113 cities in China. China’s ENGOs promote the disclosure of environmental information, promote environmental public participation, and improve the environmental governance mechanism via the coordination of specific policies, legal systems, and values. In addition, although it cannot achieve immediate effects, in the long run, environmental education can change ordinary people’s attitudes towards environmental issues, which is beneficial for environmental governance. Considering India as an example; environmental education in India has found its way into school curricula via a public interest lawsuit against environmental pollution in the Ganges river basin initiated by the famous Indian environmental lawyer and activist M.C. Mehta. In China, the government and social organizations have also paid more attention to environmental education. The organizations that provide environmental education include both the government-led environmental protection department ‘Center for environmental education and communications of Ministry of environmental protection’ and the voluntary initiative of environmental protection organizations ‘Environmental Education Association’. These organizations are involved in the process of environmental governance by organizing the public to learn about environmental protection, guiding the public to increase their environmental awareness, and leading the public to participate in environmental protection activities. Taking the Beilun District of Ningbo as an example, environmental protection and education organization guides volunteer to actively participate in media investigation and supervision activities, and they lead volunteers in key environmental enterprises and ecological areas, such as Ningsteel, North power, rock East, and Meishan Bay. Volunteers are allowed to observe the effect of ecological civilization construction and supervise the implementation of environmental laws and regulations. The environmental protection and education organization of Beilun also cooperates with the ‘green sword pioneer’ column with the Radio and Television Center. It has played a prominent role in reporting environmental news, promoting environmental protection policies, interpreting environmental laws and regulations, and spreading knowledge about ecological civilization. In addition, Beilun’s environmental protection and education organization regularly publishes the ‘Kong City Environmental Protection’ newspaper to report environmental supervision and environmental information disclosure, which has become an important force in the field of environmental governance [67]. These factors will be relevant for the future implementation of voluntary environmental policy instruments in China. In the foreseeable future, China’s voluntary environmental policy instruments will play an increasingly important role in the field of environmental governance, although the present performance is not satisfactory.

Model 4 shows that there is a negative interaction between mandatory and hybrid environmental policy instruments. Hypothesis 2 is thus proven. This confirms the findings of Doern’s [68] research. This result is mainly found because the mandatory instruments are government-led, while the hybrid instruments rely on the government’s administrative coercive power and ultimately depend on the market to be effective. Although the issue cannot be considered simply in the dichotomous perspective of the opposition between the government and the market, the excessive interference of administrative coercive power will inevitably disrupt the use of hybrid instruments that are based on the market. Hybrid environmental policy instruments provide more flexible and diversified pollution control methods for sewage treatment enterprises. Enterprises can choose to increase sewage charges or improve pollutant treatment levels, bypass the direct government-based controls, and internalize the negative externality of environmental pollution so that the entire society can achieve Pareto-optimal pollution control. The use of hybrid environmental policy instruments can break the domination of the mandatory instruments and, to a certain extent, affect the role of the mandatory instruments. Thus, there is a negative interaction between mandatory and hybrid environmental policy instruments.

Models 5 and 6 show that the degree of economic development will negatively affect the role of the mandatory instruments in promoting regional eco-efficiency. Models 7 and 8 show that the degree of economic development will positively affect the role of hybrid instruments in promoting regional eco-efficiency. Therefore, hypothesis 3 is proved. This confirms the research of Ren et al. [58] That is, in a region with a higher level of economic development, the mandatory instruments will have a weaker effect on improving eco-efficiency, while their effect will be greater in regions where the economy is less developed. In contrast, in a region with a higher level of economic development, the hybrid instruments will have a greater effect on improving eco-efficiency, while they will play a weaker role where the economy is less developed. This effect occurs because, in China, economic development is often positively related to economic freedom. The more economically developed regions are more accustomed to adopting market methods to solve problems, and the same is true in the field of environmental governance [68]. Therefore, the hybrid environmental policy instruments implemented in economically developed regions have a higher governance performance than those implemented in economically underdeveloped areas. In the economically underdeveloped regions, government administrative control has become the norm, and mandatory environmental policy instruments can often yield immediate results. In economically developed regions, the governance performance of mandatory instruments is greatly reduced. Therefore, the degree of economic development will positively affect the role of hybrid instruments in promoting regional eco-efficiency; however, it will negatively affect the role of mandatory instruments in promoting regional eco-efficiency.

## 7. Conclusions

The question of how to correctly address the relationship between economic development and environmental protection has become a difficult problem that must be faced by Chinese governments at all levels. The use of different environmental policy instruments provides a diversified solution for the government to manage environmental issues. Making a reasonable choice of environmental policy instruments is key for improving regional eco-efficiency [69].

The contribution of this paper to the research mode is to broaden the area of thought on the relationship between environmental policy instruments and eco-efficiency. Besides, the paper identifies the third-party factors that may affect the relationship of environmental policy instruments and eco-efficiency on a broader level, and then analyses the relationship furtherly. The study finds that there are mutual influences and mutual restraints among the environmental policy instruments, and the degree of economic development will also affect the performance of environmental policy instruments. Therefore, regional eco-efficiency can be improved to the greatest extent only by matching various environmental policy instruments in a manner that is suitable to the particular time and location and that realizes the complementary advantages of the instruments.

Specifically, in economically developed regions, the use of hybrid environmental policy instruments should be intensified and the use of mandatory policy instruments should be appropriately reduced. In economically developing regions, mandatory instruments must be mainly used, supplemented by hybrid instruments. Measures should be taken to reduce the high cost of policy implementation and the inefficiency resulting from the negative interaction between mandatory and hybrid instruments so that the various instruments can be most effective in improving eco-efficiency in different regions. At the same time, the intensity of the use of voluntary instruments can be strengthened in each region to involve the entire society in solving environmental problems. The Chinese government should speed up the establishment of a social participation mechanism for environmental governance. On the one hand, the government must continue to strengthen public education on the environment, strengthen the awareness of environmental protection supervision for the public, and establish incentive mechanisms for pollution reporting to increase public participation in environmental governance [70]. On the other hand, the government should strengthen cooperation with ENGO and establish an equal communication mechanism and environmental information sharing mechanism so that ENGO can play a greater role in the field of environmental governance to improve the governance performance of voluntary environmental policy instruments and regional eco-efficiency.

## Figures and Tables

**Table 1:** The direction and weight of the eco-efficiency index.

	Level Two Index	Level Three Index	Level Four Index	Unit	Weight	Index Direction
Eco-efficiency	Environmental Quality	Air	Annual Average Concentration of Sulphur Dioxide	μg/m^3^	0.0143	−
			Annual Average Concentration of Nitrogen Dioxide	μg/m^3^	0.0344	−
			Annual Average Concentration of Fine Particles (PM10)	μg/m^3^	0.0015	−
			Days When Air Quality is at or Better than Grade Two	Day	0.0100	+
		Water	Chemical Oxygen Demand (COD) Emission Intensity	kg/GDP (10,000)	0.0065	−
			Industrial Wastewater Emission Intensity	ton/GDP (10,000)	0.0129	−
		Solid Waste	General Industrial Solid Waste Emission Intensity	ton/GDP (10,000)	0.0012	−
	Eco-Protection	Biodiversity	Forest Cover Rate	%	0.0870	+
			The proportion of Nature Reserves in the Area of Jurisdiction	%	0.1278	+
		Town Greening	Greening Cover Rate of Urban Built-up Area	%	0.0152	+
			Proportion of Total Area of Wetlands in National Land Area (%)	%	0.2141	+
	Environmental Governance	Pollution Control	Rate of Industrial Waste Gas Treatment Facilities	set/MCM	0.1044	+
			Comprehensive Utilization of General Industrial Solid Waste	%	0.0097	+
			Rate of Industrial Sewage Treatment Facilities	set/10-kilotons	0.0721	+
		Environmental Regulation	Environmental Pollution Investment Intensity	%	0.2858	+
			Number of Environmental Incidents	number	0.0029	−

**Table 2:** The environmental policy instruments commonly used in China.

Mandatory Environmental Policy Instruments (MEPI)	Hybrid Environmental Policy Instruments (HEPI)	Voluntary Environmental Policy Instruments (VEPI)
‘Three-simultaneity’ institution	Sewage fee collection institution	Environmental mission
Emission discharge license institution	Emission trading institution	Environmental information disclosure institution
Pollutant emission concentration control	Environmental subsidy institution	ENGO
Deadline governance institution	Energy saving subsidy institution	Environmental impact assessment public hearing
Environmental administration inspection		Public opinion supervision
Total pollutant emission control		Green nudges

**Table 3:** The calculation method of the variables.

Category	Variable	Calculation Method	Unit
Dependent variable	EE	Entropy Weight Method	—
Independent variable	Use intensity of MEPI	Total investment in environmental protection components for projects meeting ‘three-simultaneity’ requirement/Local GDP	—
	Use intensity of HEPI	Receipt of fee on waste discharge/Local revenue	—
	Use intensity of VEPI	Number of social education activities/Local population	—
Moderator variable	PCDI	Per capita disposable income	Yuan
Control variable	Ind	Output value of secondary industry/Local GDP	%
	Investment intensity of R&D	R&D expenditure/Local GDP	—
	GDP	Gross local product	Yuan
	Pop	Local population	—

**Table 4:** The descriptive statistics of the variables in the regression analysis.

Variable	Mean	SD	Min	Max	N
lnGDP	9.08	1.08	5.52	11.12	310
ind	47.30	8.05	21.31	59.05	310
pop	0.43	0.27	0.03	0.03	310
R＆D	1.30	1.04	1.04	5.98	310
MEPI	0.38	0.26	0.03	1.92	310
HEPI	0.61	0.51	0.01	4.63	310
VEPI	0.14	0.19	0.00	2.25	310
PCDI	3.25	2.04	0.51	10.52	310
EE	34.61	9.58	21.02	100	310

**Table 5:** The regression model.

Variable	Model 1	Model 2	Model 3	Model 4	Model 5	Model 6	Model 7	Model 8
lnGDP	0.3025	1.1598	0.0974	2.0788	1.4991	2.3388	0.7419	−1.4362
ind	0.0286	−0.0141	−0.0753	−0.0112	−0.5213	−0.7555 *	−0.3358	−0.0183
pop	0.0042	0.0062	0.0011	0.0032	−0.0222	−0.0267	−0.0241	−0.0322 *
R&D	1.7289	1.3190	2.0595	2.0643	15.1598	18.0387 *	16.9950	18.1697 **
MEPI	2.1944 ***			2.0498 ***	−1.2971 ***	−3.0509 ***		
HEPI		0.7329 ***		2.4440 ***			−0.5007 *	−4.2828 ***
VEPI			1.5943					
MEPI * HEPI				−0.2307 ***				
HEPI * PCDI								0.0005 ***
MEPI * PCDI						−0.0038 ***		
PCDI					0.9213 ***	1.2078 ***	1.1397 **	1.1566 ***
Constant	27.03	22.27	34.39	10.97	34.21	35.00	29.01	27.45
N	310	310	310	310	310	310	310	310
R2	0.26	0.11	0.03	0.42	0.19	0.24	0.12	0.36
F Value	19.32 ***	7.10 ***	1.53	27.77 ***	2.48 **	2.75 **	1.42	4.96 ***
Hausman test	14.48 **	17.07 ***	18.16 ***	16.08 **	16.74 **	40.82 ***	21.74 ***	18.20 ***

Note: ***, **, and * denote significance at the 1%, 5%, and 10% levels, respectively.
